# Role of Dietary Habits in the Prevention of Diverticular Disease Complications: A Systematic Review

**DOI:** 10.3390/nu13041288

**Published:** 2021-04-14

**Authors:** Marilia Carabotti, Francesca Falangone, Rosario Cuomo, Bruno Annibale

**Affiliations:** 1 Medical-Surgical Department of Clinical Sciences and Translational Medicine, University Sapienza, 00189 Rome, Italy; marilia.carabotti@uniroma1.it (M.C.); francesca.falangone@uniroma1.it (F.F.); 2 Gastroenterology and Endoscopy Unit, “Sant’Anna e San Sebastiano” Hospital Caserta, 81100 Caserta, Italy; rcuomo@unina.it

**Keywords:** diverticulosis, colonic, diverticulitis, diet, dietary fiber, meat, alcohol drinking, coffee

## Abstract

Recent evidence showed that dietary habits play a role as risk factors for the development of diverticular complications. This systematic review aims to assess the effect of dietary habits in the prevention of diverticula complications (i.e., acute diverticulitis and diverticula bleeding) in patients with diverticula disease. PubMed and Scopus databases were searched up to 19 January 2021, 330 records were identified, and 8 articles met the eligibility criteria and were subjected to data extraction. The quality of the studies was evaluated by the Newcastle-Ottawa quality assessment form. No study meets the criteria for being a high-quality study. A high intake of fiber was associated to a decreased risk of diverticulitis or hospitalization due to diverticular disease, with a protective effect for fruits and cereal fiber, but not for vegetable fiber; whereas, a high red meat consumption and a generally Western dietary pattern were associated with an increased risk of diverticulitis. Alcohol use seemed to be associated to diverticular bleeding, but not to recurrent diverticulitis or diverticular complications. Further high-quality studies are needed to better define these associations. It is mandatory to ascertain the role of dietary habits for the development of recurrent acute diverticulitis and diverticular bleeding.

## 1. Introduction

Colonic diverticulosis is a common condition, especially in Western countries, and its prevalence increases with age, affecting up to 65% of people older than 80 years of age [1]. Although the majority of individuals with diverticulosis remain asymptomatic throughout life, approximately 15–20% of the patients will develop abdominal symptoms without overt inflammation (a condition termed symptomatic uncomplicated diverticular disease) and about 5% develop complications including acute diverticulitis or diverticular bleeding [2,3,4].

Acute diverticulitis is an inflammatory process involving one or more colonic diverticula, often associated with pericolonic inflammation and it is classified as uncomplicated or complicated (by the presence of abscesses, perforation, fistulas, obstruction, or peritonitis) and can reoccur in about 25% of cases. Differently, diverticular bleeding occurs from rupture of the intramural branches of the marginal artery at the dome or neck of the diverticulum and usually stops up to 90% of cases spontaneously; for this reason, understanding its prevalence might be difficult to establish [5].

Acute diverticulitis and diverticular bleeding appear to be unrelated diverticular complications and rarely occur together. Although the pathogenesis of diverticular complications remains poorly defined, diverticular bleeding and diverticulitis have demonstrated distinct clinical risk factors and etiopathogenesis [6,7]. Recent evidence showed that dietary habits and other modifiable lifestyle factors play an important role as risk factors for the development of diverticular complications [8]. Among dietary habits, fiber intake is the risk factor that has been most studied. Fibers are edible plant components or the analogous carbohydrates that are resistant to digestion/absorption in the small intestine, with partial or complete colonic fermentation [9]. Fiber intake, achieved by consuming fruits, vegetables, and cereal grains, increases fecal mass and regularizes bowel movements, as well as acts as a colonic prebiotic, favoring health-promoting species of the gut microbiota [10]. Previous systematic review assessed whether a high-fiber diet can improve symptoms in patients with symptomatic uncomplicated diverticular disease [11,12] or might prevent diverticular disease [13,14] however, without a clear distinction between symptomatic uncomplicated diverticular disease and diverticular complications as acute diverticulitis or diverticular bleeding. On the other hand, data regarding alcohol consumption and diverticular disease showed discordant findings, with some studies showing a positive association [15,16] and others not [17], with uncertain effects regarding coffee consumption.

This systematic review aims to assess the effect of dietary habits in the prevention of diverticula complications (i.e., acute diverticulitis and diverticula bleeding) in patients with diverticula disease.

## 2. Materials and Methods

### 2.1. Study Selection

The search was conducted according to the PRISMA (Preferred Reporting Items for Systematic Reviews and Meta-Analyses) guidelines [18]. The electronic databases PubMed MEDLINE (U.S. National Library of Medicine, Bethesda, MD, USA) and Scopus were systematically searched according to the following search strategy: (“diverticulum, colon/complications”[MeSH Terms] OR “diverticulum, colon/diet therapy”[MeSH Terms] OR “diverticulum, colon/therapy”[MeSH Terms] OR “diverticulum, colon”[All fields] OR (“diverticulosis, colonic/complications”[MeSH Terms] OR “diverticulosis, colonic/diet therapy”[MeSH Terms] OR “diverticulosis, colonic/therapy”[MeSH Terms] OR “diverticulosis, colonic”[All fields]) OR (“diverticulitis, colonic/complications”[MeSH Terms] OR “diverticulitis, colonic/diet therapy”[MeSH Terms] OR “diverticulitis, colonic/prevention and control”[MeSH Terms] OR “diverticulitis, colonic/therapy”[MeSH Terms] OR “diverticulitis, colonic”[All Fields]) OR (“diverticular diseases/complications”[MeSH Terms] OR “diverticular diseases/diet therapy”[MeSH Terms] OR “diverticular diseases/prevention and control”[MeSH Terms] OR “diverticular diseases/therapy”[MeSH Terms] OR “diverticular disease*”[All Fields])) AND (“diet/adverse effects”[MeSH Terms] OR “diet/complications”[MeSH Terms] OR “diet/deficiency”[MeSH Terms] OR “diet/prevention and control”[MeSH Terms] OR “diet/therapeutic use”[MeSH Terms] OR “diet/therapy”[MeSH Terms] OR “diet”[All Fields] OR (“dietary fiber/administration and dosage”[MeSH Terms] OR “dietary fiber/adverse effects”[MeSH Terms] OR “dietary fiber/deficiency”[MeSH Terms] OR “dietary fiber/therapeutic use”[MeSH Terms] OR “dietary fiber*”[All Fields]) OR (“meat/administration and dosage”[MeSH Terms] OR “meat/adverse effects”[MeSH Terms] OR “meat/therapeutic use”[MeSH Terms] OR “meat”[All Fields]) OR (“alcohol drinking/adverse effects”[MeSH Terms] OR “alcohol drinking/complications”[MeSH Terms] OR “alcohol drinking/prevention and control”[MeSH Terms] OR “alcohol drinking”[All Fields]) OR “coffee/adverse effects”[MeSH Terms] OR “coffee”[All Fields]) AND (English [lang]) AND (“humans”[MeSH Terms]) AND (“adult”[MeSH Terms]).

**Figure 1:**
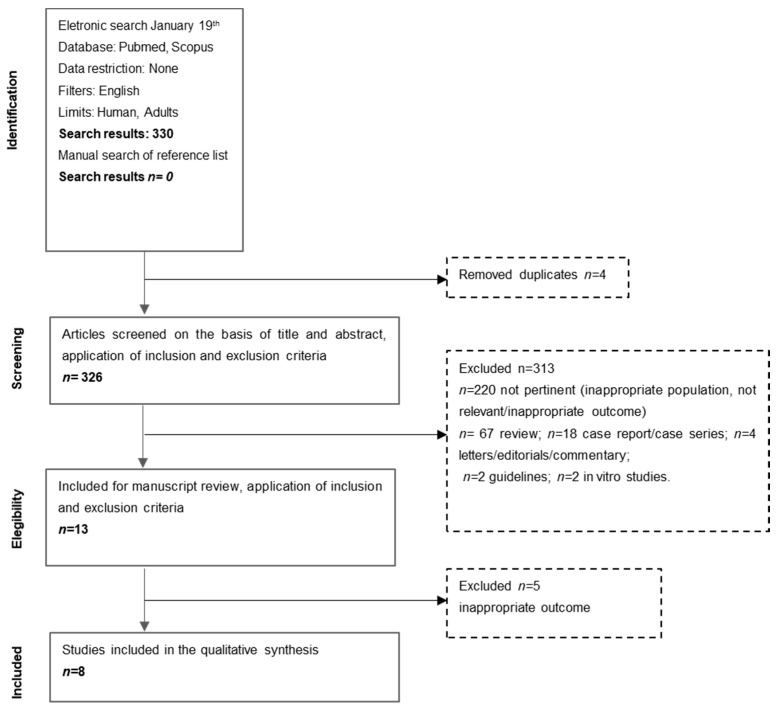
Flow-chart of study selection.

Guidelines, reviews, case reports, case series, editorials, letters, commentary, and in vitro studies were excluded. The following study types were considered: randomized controlled trials, open randomized clinical trials, non-randomized open studies, observational studies, cross-sectional studies. Pediatric subjects were excluded from the present review. No publication data restriction was imposed. Papers published in English were considered (Figure 1).

Studies were considered for inclusion in this review if they described in adult patients (>18 years) the association between dietary habits (dietary fibers, meat, coffee alcohol, and coffee consumption) and diverticular complications as acute diverticulitis or diverticular bleeding. Study methods were carefully evaluated to understand whether the outcome was based on uncomplicated diverticular disease or on documented complications as diverticulitis or bleeding. When it was not possible to distinguish between simple diverticulosis, symptomatic uncomplicated diverticular disease, and complicated diverticular disease (i.e., diverticulitis or bleeding), the paper was excluded from the analysis.

Potentially relevant articles were independently screened for eligibility in an un-blinded standardized manner by the two reviewers (M.C., F.F.), initially by title and abstract and then by full text, when necessary, in order to determine whether they met the inclusion criteria.

Disagreements between reviewers were solved by a discussion by the authors. The reference lists of the identified articles were manually searched for potential additional studies that may have been overlooked.

### 2.2. Data Extraction

A data extraction sheet has been developed using three randomly selected studies and was further refined accordingly. One review author (M.C.) extracted the data from the included studies and the second author (F.F.) checked the extracted data. Disagreements were resolved by discussion between the two authors. The following information was extracted from each included paper: (1) author, year; (2) country; (3) type of study; (4) length of follow-up; (5) number of patients, gender, and age of patients; (6) method of dietary/alcohol consumption assessment; (7) main outcome; (8) intervention and control group; and (9) main results.

### 2.3. Quality Assessment

Quality assessment of the included studies was performed using the Newcastle-Ottawa quality assessment form for cohort studies and cross-sectional studies [19] (Supplementary Table S1). Since a standard criterion for what constitutes a high-quality study has not yet been universally established, we considered a study which scored ≥7 a high-quality study.

## 3. Results

### 3.1. Search Results

The electronic search study identified a total of 330 records, 326 of which were unique (Figure 1).

The articles were screened on the basis of the title and abstract and, after application of the inclusion and exclusion criteria, 13 articles were retrieved for a full-paper evaluation [15,17,20,21,22,23,24,25,26,27,28,29,30]. Of these 13 papers, 8 met the eligibility criteria and were subjected to data extraction [20,21,22,23,24,25,26,27]. Five studies were excluded because it was not possible to distinguish between diverticulosis, symptomatic uncomplicated diverticular disease and complicated diverticular disease [15,17,28,29,30]. Thus, 8 articles were included for qualitative synthesis [20,21,22,23,24,25,26,27].

### 3.2. Characteristics of Included Studies

The main characteristics of the eight included studies are grouped into two tables: Table 1 reported data on dietary fibers and meat intake (five articles) (Table 1) and Table 2 reported on alcohol consumption (three articles) (Table 2).

### 3.3. Dietary Habits

#### 3.3.1. Dietary Fiber and Meat Consumption

Articles concerning dietary fiber and meat consumption were performed over a period of 11 years, from 2008 to 2019. All five of these studies were prospective cohort studies. Four studies were conducted in the United States [20,21,22,24] and one study was performed in the United Kingdom [23]. The follow-up protocol was variable between studies, ranging from 6 [23] to 26 years [21,22].

Regarding the patient population, three studies used the same cohort of male patients (the Health Professionals Follow-Up Study, HPFS), which included approximately 47,000 with mean age ranging from 40 to 75 years [21,22,24]; two studies were based on a women cohort including 50,019 [20] and 690,075 patients [23] respectively, with the mean age ranging from 50–65 years.

In the majority of the studies, dietary assessment was obtained from study participants using a validated semi-quantitative Food Frequency Questionnaire (FFQ) assessing the consumption of 131–148 foods and beverages [20,22,24]; in one study, the number of food items assessed through the FFQ was not reported [21]; one study was based on a questionnaire assessing the consumption of 40 common food and beverage items [23].

Two studies estimated the assumption of fiber from fruits, cereals, and vegetables and fiber from potatoes [20,23]; one study evaluated the consumption of meat (distinguishing between processed and unprocessed red meat), poultry, and fish [21]; one study identified two major dietary patterns such as a Western pattern (high intake of red and processed meats, refined grans, sweets, French fries and high-fat dairy products) and the prudent pattern (high intake of fruits, vegetables, whole grains, legumes, poultry, and fish) and calculated the Alternative Healthy Eating Index (AHEI) for each patient [22]; one study analyzed the consumption of nuts, corn, and popcorn [24].

Authors used different criteria to ascertain diverticular complications: four studies used questionnaires assessing diverticulitis through specific parameters (abdominal pain attributed to diverticula requiring hospitalization/antibiotics/surgery or complicated by perforation/abscess/fistula or obstruction, or presenting with fever, requiring medication, or evaluated using abdominal computed tomography) [21,22,24] and diverticular bleeding (rectal bleeding attributed to diverticular disease requiring hospitalization/intravenous fluids/blood transfusion/angiography/nuclear medicine scanning, or surgery; or described as profuse; or without other potential gastrointestinal, rectal, or anal sources whose bleeding was not evaluated as part of a routine endoscopy or barium study) [24]. One study was based on hospital admission code using the WHO Classification of Disease and Related Health Problems 10th Revision (ICD-10) considering a diagnosis of diverticular disease by the code ICD-10 K57, which includes diverticulitis, diverticulosis and diverticulum of the large or small intestine [23]. To try to overcome this potential bias, we limited the analysis to complicated diverticular disease (diverticula with abscess, bleeding, or perforation, ICD-10 code: K570, K572 or K578).

With regard to the intervention and control, food intake was categorized in quintiles [20,21,22,23] or detailed by frequency of food consumption (never or less than once a month, 1–3 times per month, once per week, 2–4 times per week, 5–6 times per week, once a day, 2–3 times daily, 4–5 times daily, more than 6 times a day) [24].

Looking at the main results, two studies evaluated the effect of dietary fiber and risk of diverticulitis or hospitalization due to diverticular disease: generally, a high intake of fiber was associated to a decreased risk of diverticular complications, but different effects were found when considering fiber separately from fruits, vegetables, or cereals [20,23] (Table 1). One study analyzed the association between dietary patterns and risk of incident diverticulitis, finding that a higher Western dietary pattern score was associated with an increased risk of diverticulitis, and higher prudent and AHEI dietary pattern scores were associated with a decreased risk [22]. Cao Y et al. evaluated the association between consumption of meat, distinguishing between total red meat, unprocessed (beef or lamb/pork as a main dish, hamburger, beef, pork, or lamb as a sandwich or mixed dish), processed red meat (bacon, beef, or pork hot dogs, salami, bologna, or other processed meat sandwiches and other processed red meats, such as sausage, kielbasa, etc.), total poultry consumption (chicken or turkey with or without skin, chicken or turkey hot dogs, and chicken or turkey sandwiches), and total fish intake (dark meat fish, canned tuna fish, breaded fish cakes, pieces or fish sticks, and other fish) with risk of incident diverticulitis [21]. Authors found that total red meat intake was associated with an increased risk of diverticulitis, with a non-linear plateauing after six servings per week (*p* for non- linearity = 0.002). The association was stronger for unprocessed red meat than for processed red meat. Interestingly, the substitution of poultry or fish for one serving of unprocessed red meat per day was associated with a decrease in risk of diverticulitis (multivariable RR 0.80; 95% CI 0.63 to 0.99) [21]. The last study by Strate et al. evaluated whether nut, corn or, popcorn consumption was associated with diverticulitis and diverticular bleeding. Authors found an inverse association between nut and popcorn consumption and risk of diverticulitis; no association was seen between corn consumption and diverticulitis or between nut, corn, or popcorn consumption and diverticular bleeding [24].

#### 3.3.2. Alcohol and Coffee Consumption

Articles concerning alcohol consumption were performed over a period of 20 years from 1999 to 2019 and are summarized in Table 2. One study was conducted in South Korea [25], one in Japan [26], and one in the United Kingdom [27]. One of the three selected studies was a retrospective cohort study [25], and two were cross-sectional studies [26,27]. The selected studies included a number of patients ranging from 80 to 911, with a mean age of 42–69 years. In two out of three studies, the female gender was slightly prevalent [25,27]. With regard to the alcohol consumption assessment, one study used a qualitative evaluation (yes/no) [25], one a semi-quantitative assessment (≥/< 21 units/week) [27], and another one a quantitative assessment (nondrinker/light drinker (1–180 g/week), moderate to heavy drinker (>180 g/week)) [26]. With regard to the main outcome, one study analyzed the risk factors for recurrent diverticulitis [25], one the risk factors predisposing a person to diverticular complications [27], and one diverticular bleeding [26]. Concerning the main results, the alcohol consumption seemed to be associated to diverticular bleeding [26], but no significant associations were found when considering recurrent diverticulitis [25] or diverticular complications [27].

No study was found regarding the association between coffee consumption and risk of diverticular complications.

### 3.4. Quality Assessment

Quality assessment of the included studies performed by using the Newcastle-Ottawa quality assessment form gave a mean score of 5.8 for cohort studies and 5.0 for cross-sectional studies (Supplementary Table S1). No study meets the criteria for being a high- quality study.

## 4. Discussion

This systematic review focused on dietary habits as potential risk factors for diverticular complications as acute diverticulitis or diverticular bleeding. Differently from previous systematic reviews [13,14], we tried to identify the role of different dietary components (dietary fiber, meat, alcohol, and coffee consumption) or dietetic pattern and the risk of complicated diverticular disease.

We found some interesting data. Two studies found that a high intake of fiber was associated with a decreased risk of diverticulitis [20] or hospitalization due to diverticular disease [23]. However, different effects were found when considering the source of dietary fiber with a protective effect for fruits and cereal fiber, but not for vegetable fiber [20,23]. The inverse association between fiber intake and risk of diverticular disease was also reported by other authors [17,29]. Aldoori et al. found that high intake of dietary fiber reduces the risk of diverticular disease and suggests that the inverse relation is particularly strong for the insoluble component of fiber and most notably for cellulose [29]. Similarly, Crowe et al. also found an inverse association with dietary fiber intake: participants in the highest quintile (≥25.5 g/day for women and ≥26.1 g/day for men) had a 41% lower risk of diverticular disease (RR = 0.59, 0.46 to 0.78) compared with those in the lowest quintile (<14 g/day for both women and men) [17]. Another recent study by Mahmood et al. included two parallel cohorts of women (n = 36,110) and men (n = 44,723) followed for seven years; authors reported that a high intake of fruits and vegetables may reduce the risk of hospitalization due to diverticular disease, but intake of cereals did not influence the risk [28]. However, in these studies, it was not possible to distinguish between diverticulitis, diverticular bleeding, and symptomatic uncomplicated diverticular disease [17,28,29].

Regarding specific food, an interesting study by Strate et al. evaluated the association between nut, corn, and popcorn consumption and incident acute diverticulitis and diverticular bleeding. Results from this study radically changed common beliefs that recommended avoiding nuts, seeds, popcorn, corn, and other high-residue foods in order to reduce the risk of diverticular complications. Contrary to historical physician advice, authors found an inverse association between nut and popcorn consumption and the risk of diverticulitis, but no association was seen between corn consumption and diverticulitis or between nut, corn, or popcorn consumption and diverticular bleeding [24]. Another study by Strate et al. analyzed the dietary patterns of a large cohort of men followed for 26 years: the Western type of diet (high intake of red and processed meats, refined grans, sweets, French fries, and high-fat dairy products) was positively associated with increased risk of diverticulitis compared to a prudent diet (high intake of fruits, vegetables, whole grains, legumes, poultry, and fish), suggesting that beyond the single food, the attention must be focused on the entirety of dietary pattern [22].

Comparing these studies, we would like to underline some important points. First, the majority of these studies focus the attention on acute diverticulitis, whereas fewer data are available concerning diverticular bleeding; consequently, the warning to increase fiber intake could be useful for the prevention of diverticulitis, but the effects on bleeding are uncertain. Second, the protective effects shown for the prevention of the first episode of diverticulitis may not be the same as those useful for the prevention of recurrent diverticulitis. In fact, as also suggested by the recent guidelines from the European society of Coloproctology, a high-fiber diet may be recommendable for general health purposes, but there is little evidence that it can prevent recurrent diverticulitis [31].

Interesting results were obtained when the effect of meat consumption was evaluated. Cao et al. found that intake of red meat, particularly unprocessed red meat, was associated with an increased risk of diverticulitis in a prospective Health Professionals Follow-Up Study of 46,461 men; moreover, substitution of unprocessed red meat with poultry or fish may reduce the risk of diverticulitis [21]. The mechanisms through which red meat consumption may influence the risk of diverticulitis remain to be established. It is likely that the increased consumption of meat might be associated with higher levels of inflammatory bio-markers and chronic diseases and might lead to alterations of the gut microbiota favoring a pro-inflammatory state [32,33,34].

We found discordant evidence regarding alcohol consumption and diverticular complications: alcohol use seemed to be associated with diverticular bleeding [26], but no significant associations were found when considering recurrent diverticulitis [25] or diverticular complications [27]. We believe that some factors may explain these disagreements: (i) it is possible that alcohol consumption might predispose a person to diverticular bleeding, but not to acute diverticulitis; (ii) these studies belong to Western and Eastern patient cohorts and we cannot exclude that the effect of alcohol may be different considering right and left diverticulosis; (iii) alcohol consumption assessment is different between studies (qualitative/semi-quantitative and quantitative); (iv) the diagnosis of diverticula complications is made through different methods, being in some cases not fully adequate (Kim et al. diagnosed acute diverticulitis even with colonoscopy); (v) risk factors regarding primary prevention (progression from diverticulosis to diverticulitis) and secondary prevention (recurrent diverticulitis) may be different. Other studies that were not included in this review (because it was not possible to distinguish between diverticulitis, diverticular bleeding, and symptomatic uncomplicated diverticular disease) evaluated the association between alcohol use and diverticular disease. In a study by Aldoori et al. of the large prospective HPFS cohort, alcohol intake (comparing men who drink >30 g of alcohol/day to nondrinkers) was only weekly and non-significantly associated with risk of symptomatic diverticular disease (RR = 1.36; 95%CI: 0.94 to 1.97) [15]. Similarly, in the EPIC-Study, after adjusting for smoking, there was no significant association between the consumption of alcohol and risk of diverticular disease [17]. No effect of alcohol use was also found in a recent multicenter cohort study, conducted on 1217 patients, and evaluating differences between patients with previous diverticulitis and diverticula bleeding [7].

Finally, our systematic review did not find any paper assessing the effect of coffee consumption and the prevention of diverticula complications; so, the effect of this beverage is unknown.

Regarding the quality of studies, no study meets the criteria for being a high-quality study (mean score of 5.8 for cohort studies and 5.0 for cross-sectional studies). Factors that mostly contributed to lower the quality of the studies were the limited generalizability, the self-report of the exposure, and outcomes; on the other hand, one of the strengths was the length of the follow-up, long enough for outcomes to occur.

Thus, although data emerging from this systematic review are interesting, we have to bear in mind that the included studies present significant limitations, particularly the representation of the exposed cohort, the ascertainment of dietary habits, and outcome, influencing their generalizability and result translation in clinical practice. Furthermore, we must underline that the relatively small effect sizes reported might be associated with small changes in risk of developing diverticular complications. Most of these studies looked at healthy populations followed forward in time; consequently, data should be interpreted with caution and avoid providing advice that is not based on strong evidence in this setting. In addition, since available data come from the US, UK, and Asia, we cannot exclude that different results might come from countries where dietary habits are different, for example, Southern Europe where the Mediterranean diet is widespread.

Further, well-designed studies, are needed to fully understand the possible role of dietary habits in the prevention of diverticular complications, particularly regarding the risk of diverticular bleeding and recurrent diverticulitis.

## 5. Conclusions

Even if no study meets the criteria for being a high-quality study, we can conclude that a high intake of fiber was associated with a decreased risk of diverticulitis or hospitalization due to diverticular disease; whereas, a high red meat consumption and a general Western dietary pattern was associated with an increased risk of diverticulitis, and conflicting results regarding alcohol use. Further high-quality studies are needed to better define these associations. It is mandatory to ascertain the role of dietary habits for the development of recurrent acute diverticulitis and diverticular bleeding.

## Figures and Tables

**Table 1:** Effect of fiber and meat consumption in the prevention of acute diverticulitis and diverticula bleeding.

Author, Year	Country	Type of Study	Follow-Up (Years)	Number of pts, Gender, Age	Dietary Assessment	Main Outcome	Intervention/Control	Main Results
Ma W, 2019 [20]	US	Prospective cohort study (Nurses’ Health Stydy)	24	50,019 women, 43–70 years	FFQ (131 food items)	Dietary fibre consumption and risk of diverticulitis	Intake of fiber divided into quintile	**Diverticulitis** Compared with patients in the lowest quintile (Q1), patients in the highest quintile (Q5) had: Multivariable adjusted HR = 0.86 (95%CI: 0.78–0.95) for total fibre; HR = 0.89 (95%CI: 0.81–0.99) for cereal fibre; HR = 0.83 (95%CI: 0.75–0.92) for fruit fibre; HR = 0.91 (95%CI: 0.83–1.01) for vegetable fibre.
Cao Y, 2018 [21]	US	Prospective cohort study, (Health Professional Follow-up Study)	26	46,461 men 40–75 years	FFQ	Meat consumption and risk of diverticulitis	Intake of meat divided into quintile	**Diverticulitis** Compared with men in the lowest quintile (Q1), men in the highest quintile (Q5) had: Multivariable RR of 1.58 (95% CI: 1.19–2.11) for total red meat; Multivariable RR of 1.51 (95% CI: 1.12–2.03) for unprocessed red meat; Multivariable RR of 1.03 (95% CI: 0.78–1.35) for processed red meat. Higher consumption of poultry or fish was not associated with risk of diverticulitis.
Strate LL, 2017 [22]	US	Prospective cohort study, (Health Professional Follow-up Study)	26	46,295 men 40–75 years	FFQ (131–148 food items)	Dietary patterns and risk of diverticulitis	Western and Prudent dietary pattern, AHEI, into quintile	**Diverticulitis** Compared with men in the lowest quintile (Q1), men in the highest quintile (Q5) had: Multivariate HR= 1.55 (95% CI:1.20–1.99) for Western pattern; Multivariate HR = 0.74 (95%CI: 0.60–0.91) for Prudent pattern; Multivariate HR = 0.67 (0.55–0.82) for AHEI pattern.
Crowe FL, 2014 [23]	UK	Prospective cohort study (The Million Women Study)	6	690,075 women 50–65 yrs	Questionnaire (40 food and beverage items)	Dietary fibre consumption and hospitalization for diverticular disease	Intake in quintile of fiber (g/day)	Complicated diverticular disease * The RR for complicated DD per 5 gr/day were: Total fiber: 0.70 (95%CI: 0.58–0.84); Cereal: 0.65 (95%CI: 0.49–0.85); Fruit:0.60 (95%CI: 0.42–0.85); Non-potato vegetable: 0.70 (95%CI: 0.37–1.30).
Strate LL, 2008 [24]	US	Prospective cohort study, (Health Professional Follow-up Study)	18	47,228 men 40–75 years	FFQ (131 food items)	Nut, corn and popcorn consumption and risk of diverticulitis or diverticular bleeding	Frequency of food consumption, (from never /less than once a month, to more than 6 times a day)	**Diverticulitis** Compared to men with the highest intake, men with the lowest intake had: Multivariate HR: 0.80 (95%CI: 0.63–1.01) for nuts; Multivariate HR: 0.72 (95% CI: 0.56–0.92) for popcorn; Multivariate HR: 1.13 (95% CI: 0.83–1.54) for corn. **Diverticular bleeding** Multivariate HR: 1.08 (95%CI: 0.77–1.49) for nuts; Multivariate HR: 0.82 (95% CI: 0.59–1.15) for popcorn; Multivariate HR: 1.07 (95% CI: 0.67–1.71) for corn.

Legend: UK: United Kingdom; Pts: patients; FFQ: food frequency questionnaire; HR: hazard ratio; RR: relative risk; Alternative Healthy Eating Index (AHEI); * complicated diverticular disease was defined as diverticula with abscess, bleeding, or perforation (ICD-10 = code: K570, K572, K578).

**Table 2:** Effect of alcohol consumption in the prevention of acute diverticulitis and diverticula bleeding.

Author, Year	Country	Type of Study	Follow-Up (Months)	Number of pts, Gender, Age	Alcohol Consumption Assessment	Main Outcome	Intervention/Control	Main Results
Kim YC, 2019 [25]	South Korea	Retrospective cohort study	32.9	296 pts; women 58.1%; mean age 42.8 ± 13.7 years	Qualitative assessment	Risk factors for recurrent right colonic diverticulitis	Alcohol consumer vs non-alcohol consumer	**Recurrent diverticulitis** Recurrence rate was higher in alcohol consumer than non-alcohol consumer (*p* = 0.017). The association was not confirmed at multivariate analysis.
Nagata N, 2014 [26]	Japan	Cross-sectional study	NA	911 pts; women 34%; mean age 66 ± 12 years	Questionnaire	Risk factors for diverticular bleeding	Quantitative assessment: nondrinker/ light drinker, moderate to heavy drinker	**Diverticular bleeding** Multivariable adjusted HR: OR 3.4 (95%CI:1.4–8.1) for light drinker Multivariable adjusted HR: OR 3.3 (95%CI 1.3–8.5) for moderate to heavy drinker.
Papagrigoriadis S, 1999 [27]	UK	Cross-sectional study	NA	80 pts; women 62.5%; Mean age 69.1 years	Clinical assessment	Risk factors for diverticular complications	Alcohol consumer (≥21 units/week) vs non-alcohol consumer	**Complicated diverticular disease (diverticulitis and bleeding)** Alcohol consuption was not significantly more common in group 1 (complicated diverticular disease) than in group 2 (not complicated diverticular disease) (OR 2.7, 95%CI: 0.9–7.7)

Legend: pts: patients; UK: United Kingdom; NA: not applicable.

## Data Availability

Data sharing not applicable.

## Supplementary Materials

The following are available online at https://www.mdpi.com/article/10.3390/nu13041288/s1, Table S1: Quality assessment according to Newcastle Quality Assessment Scale.
