# Supplementary material for: Role of Dietary Habits in the Prevention of Diverticular Disease Complications: A Systematic Review
Source: Nutrients. 2021 Apr 14;13(4):1288. doi: 10.3390/nu13041288 (PMC8070710; doi:10.3390/nu13041288)
Supplement: Supplementary file 1 [file nutrients-13-01288-s001.pdf]

Supplementary Table S1. Quality assessment according to Newcastle Quality Assessment Scale

Newcastle Quality Assessment Scale- COHORT STUDIES

	Selection				Comparability	Outcome			
**Study**	**Representativeness of the exposed cohort**	**Selection of the non exposed cohort**	**Ascertainment of exposure**	**Demonstration that outcome of interest was not present at start of study**	**Comparability of cohorts on the basis of the design or analysis**	**Assessment of outcome**	**Was follow-up long enough for outcomes to occur**	**Adequacy of follow up of cohorts**	**Total ***
Ma W, 2019	0	1	0	1	2	0	1	1	6
Cao Y, 2018	0	1	0	1	2	0	1	1	6
Strate LL, 2017	0	1	0	1	2	0	1	1	6
Crowe FL, 2014	0	1	0	1	2	1	1	Unclear	6
Strate LL, 2008	0	1	0	1	2	0	1	1	6
Kim YC, 2019	1	1	Unclear	1	1	Unclear	1	Unclear	5

Newcastle Quality Assessment Scale- CROSS-SECTIONAL STUDIES

**Study**	**Selection**				**Comparability**	**Outcome**			
	**Representativeness of the sample**	**Sample size**	**Non-respondents**	**Ascertainment of the exposure**	**The subjects in different outcome groups are comparable, based on the study design or analysis. Confounding factors are controlled.**	**Assessment of the outcome:**	**Statistical test**	**Total ****	
Nagata N, 2014	1	0	Unclear	1	1	2	1	6	
Papagrigoriadis S,1999	0	0	Unclear	1	1	1	1	4	

Legend of the table:

1= Adequate; 0= Inadequate; Unclear

*the maximum is 9

**the maximum is 10
